# FROM SHARING TO SELLING: CHALLENGES AND OPPORTUNITIES OF ESTABLISHING A DIGITAL HEALTH DATA MARKETPLACE USING BLOCKCHAIN TECHNOLOGIES

**DOI:** 10.30953/bhty.v5.184

**Published:** 2022-01-28

**Authors:** Mohamed A. Maher, Imtiaz A. Khan

**Affiliations:** 1 Cardiff School of Technologies, Cardiff Metropolitan University, Cardiff, United Kingdom; 2 Balsamee LTD, Cardiff, United Kingdom

**Keywords:** blockchain, EHR, marketplace, GDPR, general data protection regulation, incentives

## Abstract

During the COVID-19 pandemic, we witnessed how sharing of biological and biomedical data facilitated researchers, medical practitioners, and policymakers to tackle the pandemic on a global scale. Despite the growing use of electronic health records (EHRs) by medical practitioners and wearable digital gadgets by individuals, 80% of health and medical data remain unused, adding little value to the work of researchers and medical practitioners. Legislative constraints related to health data sharing, centralized siloed design of traditional data management systems, and most importantly, lack of incentivization models are thought to be the underpinning bottlenecks for sharing health data.

With the advent of the General Data Protection Regulation (GDPR) of the European Union (EU) and the development of technologies like blockchain and distributed ledger technologies (DLTs), it is now possible to create a new paradigm of data sharing by changing the incentivization model from current authoritative or altruistic form to a shared economic model where financial incentivization will be the main driver for data sharing. This can be achieved by setting up a digital health data marketplace (DHDM).

Here, we review papers that proposed technical models or implemented frameworks that use blockchain-like technologies for health data. We seek to understand and compare different technical challenges associated with implementing and optimizing the DHDM operation outlined in these articles. We also examine legal limitations in the context of the EU and other countries such as the USA to accommodate any compliance requirement for such a marketplace. Last but not least, we review papers that investigated the short-, medium-, and long-term socioeconomic impact of such a marketplace on a wide range of stakeholders.

Since the early introduction of digital health, information and communications technology (ICT) developers have been under the impression that the use of digital technology in handling and processing health information will generate a wealth of data that can transform the healthcare industry. Feeding these data to machine learning algorithms will enable us to de-skill medical practice and propose new diagnostics and treatment processes. Projects like DeepMind Health is a recent example where an artificial intelligence company based in London and owned by Alphabet developed mobile app streams (1) that use London Royal Free hospital’s EHR data to predict and identify patients about to get acute kidney injury—a condition linked to 100,000 deaths in the UK every year (2). In addition, portals like PatientsLikeMe (3) through which patients with similar medical conditions and/or concerns can share information regarding their treatments and have demonstrable benefits to their users (4, 5).

As these projects started to show the value of data sharing, the General Data Protection Regulation (GDPR) (6) of the European Union (EU), introduced in 2018, has fundamentally changed the paradigm of sharing and using patient data by repositioning ownership and stewardship of medical data from service provider to the patient, along with bestowing the following rights as listed in Table 1.

**Table 1:** General data protection regulation of the EU fundamentally changed the paradigm of sharing and using patient data by repositioning ownership and stewardship of medical data from service providers to the patient, along with bestowing the following rights (6)

General data protection regulation	Defined
GDPR Art 12 and 13	The right to be informed • Individuals’ right to be informed about the collection and any usage of their data.
GDPR Art 15	The right of access: • Individuals have the right to access their data.
GDPR Art 16	The right to rectification: • Individuals’ right to have not correct personal data amended or completed if it was incomplete.
GDPR Art 17	The right to erasure: • Individuals’ right to have personal data erased, that is, usually called “the right to be forgotten.”
GDRP Art 18	The right to restrict processing: • Individuals’ right to request the restriction or suppression of their data.
GDPR Art 20	The right to data portability • Allows individuals to carry, move, copy, or send their data easily from one IT system to another safely and securely, without affecting its usability.
GDPR Art 21	The right to object: • Individuals have the right to object to the processing of their data in certain circumstances.
GDPR Art 22	Rights concerning automated decision-making and profiling: • Rules to protect individuals, if an organization is carrying out automated decision-making that has significant effects on them

Had DeepMind not initiated before the introduction of GDPR or PatientsLikeMe were within the European Economic Area (EEA) jurisdiction, neither of the aforementioned projects could even be initiated. It is now more evident that the data have a single owner and gatekeeper regarding access or distribution rights. Even anonymously, only the patient has the right to grant access to their data and allow using the information in a way that will benefit everybody. Ironically, the patients have not yet recognized the value of this new ownership right and how to manage its stewardship. This is because there are no direct wins created to serve them, as well as indirect wins, which are not clear enough to overcome the legitimate worries of data leakage and subsequent privacy exposure. Therefore, the target is to find a novel approach and tools that balance individual privacy and transparent data access for research purposes (7).

This paradigm shift introduced by GDPR to the service providers also brought opportunities for individuals to monetize their medical data by selling data to medical researchers or tech companies. Similar to Airbnb, which enabled individuals to monetize their spare accommodations, patients with their new ownership and other rights bestowed by GDPR, can now monetize their personal health data through a “Digital Health Data Marketplace” (DHDM) using a shared economic model. Figure 1 illustrates the DHDM operational workflow. However, with the current centralized data management framework where EHRs are fragmented across different service providers where regulations differ across organizations and geographical jurisdictions, access and stewardship will be challenging to manage, especially the microtransactions in such a distributed environment. In this context, blockchain and associated smart contracts have been considered a game-changing technology, with an inbuilt distributed architecture and the ability to administer information governance in a decentralized manner for diverse types of transaction-based digital services.

**Figure 1:**
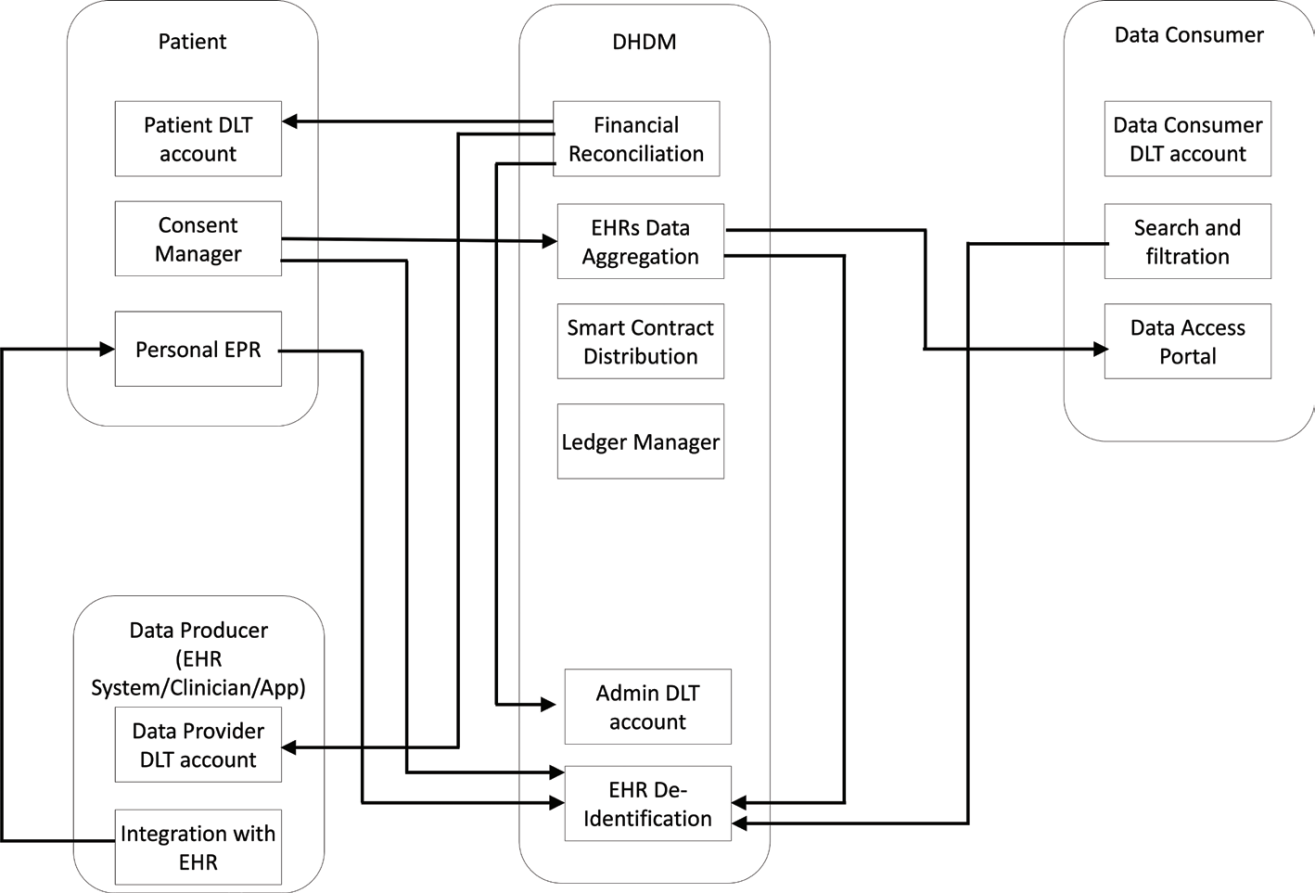
Four interfaces of the operational workflow of DHDM: Through the patient interface (top left) the patient sets their accounts, completes their electronic health record (EHR), and manages access control via giving consent to sharing their data with researchers of their choice. Through the data producer interface (bottom left), care providers and other data producers can create their accounts and manage linking patients to their local files through the caregiver’s information technology system. Researchers will set up their accounts, search for datasets, request access to data, and make payments for the data they access through the data consumer interface (top right). The back-end administration of the marketplace will be carried out through the DHDM interface, EPR (electronic patient record) and DLT (distributed ledger technologies).

The rest of the paper is organized as follows: first, we reviewed 36 papers concerning the technical challenges, focusing on three areas such as data ownership and access control, data interoperability, and data security. Then, we reviewed seven papers concerning legal issues, and finally, nine papers related to socioeconomic issues.

## TECHNICAL CHALLENGES

### DATA OWNERSHIP AND ACCESS CONTROL

Who owns the healthcare data? A question that has always been intriguing and creating debates from a technical, legal, and philosophical perspective. Kostkova et al. have argued the subject and questioned if health data should be open for research use in an attempt to balance between individuals’ privacy and the value of data-backed research on the life of millions around the world (7). The authors concluded by urging policymakers at the international level to develop a regulatory framework that safeguards personal information, limits business exploitations, yet enables the use of data for research and commercial use.

A significant amount of work has been done in proposing blockchain-based access control tools and models, which places the patient in the driver seat and gives them all the control to grant and deny access to part or the whole of their EHR. Most of the studies were trying to give such control to the patient for clinical and operational benefits. However, the same proposed models can also benefit data control from an asset management perspective. Bahar et al. offer a specific literature review covering most of the work in this domain. In this survey, the authors mainly covered and discussed work covering digital identity records management and the self-sovereignty of EHR data (8). They produced a list of social data solutions implemented using Ethereum smart contracts and compared them based on incentive, data market, enabling PHR, decentralized asset tracking, Web/Mobile application, IoT, EHR compatibility/interoperability, and proof-of-concept implementation.

As discussed above, Nguyen presented a model for secure access control for EHR stored in an InterPlanatery File System (IPFS) configuration (9). The model proposed an EHR manager based on a smart contract to administer access and data transactions requests while providing the patient with a blockchain interface-based mobile app to exercise their rights to control access. Although this model might be a fascinating solution tackling the decentralization nature of EHR and yet providing secure and traceable tools for data access control and entry audit trail, all the practical trials of this configuration showed very high latency in operation. Rifi et al. addressed the same concept of using blockchain to administer transactions in EHR(10). The authors handled the control of data acquired from personal medical devices and sensors and proposed a DApp eHealth blockchain to control read/write in the EHR database being cloud or IPFS.

Nortey et al. offer another example of a blockchain framework proposal for EHR privacy management by giving patients control over who accesses their EHRs (11). The authors introduced a channeling mechanism that ensures that patients authorize entities within the distributed network to access their information. Others take a different approach, in which the authors aimed to build a consent model for data sharing (12, 13). They proposed a transactions workflow and created Ethereum smart contracts based on LUCE (14). A blockchain solution for monitoring data License accoUntability and ComplianceE and followed on it by building a consent-based architectural model then implemented it on D1NAMO data sets (15) of 29 participants. In addition, it is proposed a semi-decentralized approach as the permissioned blockchain network is distributed across organizations (13). The access control rules are coded into smart contracts that are distributed across the blockchain network. The same path is presented by Guo et al. but proposed a hybrid Blockchain-Edge Architecture (16). EHR data is stored on edge nodes that impose attribute-based access control policies. The authors used the Hyperledger Composer Fabric blockchain programmed with smart contracts and access control lists policies to evaluate the performance by measuring the transaction processing and response time against unauthorized retrieval attempts. The experiments showed that the system provides results in milliseconds, making it suitable to be incorporated in real time and secured EHR data access control frameworks. The most significant result is that the implementation showed consistency over the different sizes in all trials. This result indicates that this architecture could be the most scalable model presented in EHR consent management.

Others also provided attribute-based blockchain signature models to achieve confidentiality, integrity, and authentication of the patient data while supporting data sharing between concerned parties (17–20). Seol et al. prepared their study in a way that sees the model from the view of different policymakers and built their model in two stages (access control and digital signature), allowing smart contracts to impose each policy rule in turn (20). Yang et al. (21). built on the attribute-based model by Wang et al. (17) and built a demonstration to measure performance, especially encryption and search time, and proved that time was independent of the number of attributes.

Guang et al. provided a model that depends on the care provider to control the EHR transactions (22). What is interesting in this model is that the authors propose an architecture that implements blockchain technology with the existing EHR system. Considering that an EHR system has to have a multiple access system and that health providers individually maintain records as per the authors’ process design, the model gave providers primary responsibilities, including creating, verifying, and appending new blocks. The design uses smart contracts, where this architecture is independent of any specific blockchain platforms, and its variations can potentially apply to any EHR system.

### DATA INTEROPERABILITY

Data interoperability is one of the critical challenges for health informatics due to the heterogeneous nature of the data and the lack of standardization in different EHR systems.

MedRec (23) was the base that many researches in blockchain used to securely exchange/transfer data from distributed systems into a unified patient EHR. MedRec issued an industrial white paper that explains an open-source blockchain model to handle the secure transfer of EHR data entries from healthcare provider systems to patient nodes and vice versa. The aim is to securely collect the data created in a local patient file at any number of hospitals and aggregate them in a consolidated file under the patient’s control. Being open source, encouraged many researchers to use it in trial implementations and similarly encouraged industrial pilots to adopt their model. This white paper model is one of the very few blockchains in healthcare models that have been implemented. The work by Yang et al. is an example of an academic build upon the MedRec framework (24).

MedShare (25) is one of the early proposed EHR data exchange control models using blockchain. The authors started by suggesting a processing layer to administer the exchange of information between existing healthcare providers’ cloud infrastructure. However, the simulation showed that latency is relatively high and increases with the increase in the number of users. MedBlock (26) is a similar model. Here the authors proposed to use nontraditional blockchain entities, such as authentication servers and Certificate Authorities, to provide means to issue identities and secure the cryptographic material, which will be used to encrypt all data on the blockchain. Although MedBloc was designed to match the healthcare IT infrastructure in New Zealand, the researcher could not spot a uniqueness that would hinder its implementation elsewhere.

Xiaoguang et al. (27) presented a recent adaptation of the MedRec model. This time the aim was to provide and implement a tamper-resistant medical data sharing scheme—a Delegated Proof of State mechanism to act as the lightweight and reliable consensus mechanism. The analysis results proved the scheme satisfactory and had a low computational and communication cost. This scheme is a perfect match to the scope of the data marketplace research, except that it is a non-payment scheme.

Zhuang et al. (28) provided another framework that differs from the MedRec model. Although it targets the same purpose, to achieve patient-centric health information exchange, this framework focused on empowering patient control with tools. The framework then created a DApp for the patient where they can adjust parameters in the smart contracts by giving permissions, allowing touchpoints, and managing access requests through linkage and request modules. This framework offers practical traits to the system: a blockchain adapter set up for communication, sending/receiving healthcare records, and create a graphical presentation for users with easy interaction, two security layers to ensure only authorized smart contract functions execution, minimize the risk of a data breach, hashing for data consistency, data segmentation that allows partial data sharing, and touchpoint selection for clinicians to select the relevant data segment to the specialty.

### DATA SECURITY

De-identify the patient record is fundamental to ensure privacy and security. This needs to be tackled on two fronts in parallel. One is segregating the identifiable patient parameters from the clinical data. Segregation must be done in the application, communication, and storage layers. The other is in the clinical data itself. For example, by design, any digital imaging and communications in medicine (DICOM) image will have identifiable data such as patient name, date of birth, the referring body. Therefore, de-identification and anonymization must be carried out before placing the data on an immutable blockchain network.

Several researches adopted the model of storing the EHR in blockchain (29, 30) This approach was discounted over time for technical and legal reasons. Technically, this was because of the size of the block and the capacity to store a large amount of data in a chain that is replicated over many nodes. Legally, it barely adheres to the requirements of GDPR Article 17 (6) concerning the right widely known as the right to be forgotten, as it is not possible to amend or delete a record once it is stored on the chain. One of the studies that adopted the EHR on the chain approach is by Tang et al. (30). Naturally, it would not have been of relevance to this research. However, the authors proposed an interesting model for authentication by designing an identity-based signature scheme with multiple authorities for the blockchain-based EHR system. The scheme offers what could be efficient signing and verification algorithms.

A large number of publications proposed what has mostly been referred to as cloud-assisted blockchain EHR security. Wang et al. (31) presented a cloud-assisted secure and privacy-preserving EHR sharing protocol based on a consortium blockchain. In other words, EHR is stored on the cloud while EHR indexes (log keeping) are kept on the blockchain. In their work, the authors proposed a blockchain-based EHR sharing scheme with conjunctive keyword searchable encryption and conditional proxy re-encryption to realize data security and privacy preservation of data sharing between different medical organizations.

In addition, Kim et al. (32) provided a model and a simulated trial for a secure protocol for a cloud-assisted EHR system using blockchain. They demonstrated the safety of the proposed scheme against man-in-the-middle (MITM) and replay attacks using automated validation of internet security protocols and applications (AVISPA) simulation. Similarly, Vora et al. (33) proposed a model that uses blockchain to enhance the security of EHR databases. Here the authors capitalized on Ethereum smart contracts to manage consensus, permissions, classifications, and services. The model looks promising and suggested six algorithms to address transaction security and privacy preservation. Nevertheless, the model has shown that it would be implausible to completely hide all information and maintain an accessible and interoperable system. However, by using smart contracts to separate information, the proposed model still offers significant privacy preservation and data integrity. Furthermore, with a smart contract, one can determine the information access level, but in public blockchain, integration with the smart contract is challenging and not practical.

Although being a Hungarian study, Magyar et al. (34) presented a blockchain signature-based model that adopts the American Health Insurance Portability and Accountability Act (HIPAA) regulations. The model uses smart contracts and the innovations of the cryptography industry, blind signatures, multisignatures, hierarchical signatures, and other security procedures that ensure access to the information. At the same time, on the route, no one can read any open text data.

The above studies dealt with the EHR as a single database, either local or cloud stored, and discussed different approaches to using blockchain to securely adding, deleting, and modifying entries in the EHR. However, one of the main reasons why blockchain is identified as a potential technology to increase the robustness of EHR and its related transaction is that EHRs by nature are decentralized. A typical patient will have different EHRs at primary, secondary, and tertiary care. Just these three levels over a patient’s lifetime can generate tens of thousands of records that need to be combined together to form a whole patient EHR.

In contrast, Ayesha et al. (35) discussed an alternative architecture that also challenged the principle of EHR storage on the cloud. The authors suggested a framework that proposes measures to ensure the system tackles the problem of data storage as it utilizes the off-chain storage mechanism of the IPFS. Their paper evaluates the performance of the different topologies over execution time, throughput, and latency. It proposes a framework that is a combination of secure record storage along with blockchain access rules for EHRs.

Another model by Nguyen et al. (9) targets secure access control for EHR that also proposes an InterPlanetary File System (IPFS) configuration for the EHR storage. The idea is to form an IPFS node at each care provider and create an EHR manager (Server) that will play the role initially played by cloud EHR. The model then uses blockchain to index the transactions trail and deal with the EHR manager as the cloud service. Internally, the EHR manager is responsible for aggregating the patient record from all IPFS nodes upon request and create more nodes as the patient moves between different care providers. The model suggests that the EHR manager itself be based on a smart contract to administer access and data transactions requests while providing the patient with a blockchain interface mobile app to exercise their rights to control the access.

### LEGAL AND ETHICAL CHALLENGES

The legal argument is always started by who owns the data? Ownership is often confused with access. Kostkova et al. (7) aimed to distinguish between data ownership and right of access and finding novel balanced approaches to satisfy business interests and actively engage the public while securing transparent data access for research needs and large-scale integrations preserving individual privacy.

A study by Castillo et al. (36) works to identify barriers for information exchange within the context of The Health Information Technology for Economic and Clinical Health (HITECH) Act to create a more efficient and effective healthcare system. The findings suggest that a hospital is more likely to be exchanging clinical summaries with hospitals outside its health system when the other hospital uses the same EHR vendor. The authors highlight the importance of EHR vendor neutrality and thus the importance of EHR systems interoperability.

In a critical survey by Yadav et al. (37) about mining clinical data from patient’s EHR, the authors explore, discuss, and present novel insights on how data mining techniques have been utilized for EHRs. In this systematic review, they discuss application, study design, and data mining methodology of a large number of initiatives for clinical data mining. Furthermore, the authors discuss the barrier to the widespread use of data mining in clinical practice. The review itself does not cover the DHDM legal and regulatory needs. However, it tackles the ethics and compliance of the data mining research (AI, ML, etc.) facilitated through the DHDM.

Mello Michellem presented a comprehensive manual for the barriers to the growth in health data exchange within the context of the North American laws (38). The authors analyzed the federal and state health information privacy statutes and regulations and secondary materials then concluded that some critical legal barriers persist, but many issues that care providers acknowledge as obstacles are somewhat illusory. The authors emphasized that healthcare providers perceive health information privacy laws to be obstructing the growth of electronic health data exchange and blamed several factors such as the inconsistency in the patient consents laws, the special treatment for sensitive health data, and failure to establish a unified patient indexing system.

A techno-regulatory document compares differences in health data transmission standards (ISO/IEEE 11073, IHE PCD-01, and HL7 DoF) and suggests the most suitable environment to use each of them (39). The authors conclude that ISO/IEEE 11073 messages cannot contain patient information, IHE PCD-01 messages have limited device information, and that HL7 DoF has the most comprehensive information coverage in all four parameters of the study (human readability, learnability, implementation, and extensibility).

## SOCIOECONOMIC CHALLENGES

The term “creative destruction” coined by Joseph Schumpeter explains how the process of industry transformation revolutionizes the economic structure from within by destroying the existing one and simultaneously creating a new one (40). With disruptive technologies like blockchain and industries like health care, the structure is extremely complicated in terms of stakeholder engagements and economic impetuses.

Although nonmedical, the model presented by a study (41) provided a promising marketplace implementation based on an existing model used for commercial vehicles data marketplace from Japan. ID-Link was a successful model when the government of Japan initiated the construction of an information infrastructure to share data in different business areas. One of these areas was sharing data from individual EHR. In their paper, the authors replace automotive data such as speed, time, range, emission, and so with medical data from the EHR. The study discussed engagement options (Opt-In vs. Opt-Out), access control privileges, and data standardization, especially adopting specific formats such as HL7 (Paper suggested V2.5., however, FHIR HL7-V3.0 is currently widely in use all over the world), WHO ICD-10, and SNOMED-CT as a clinical terminology library. The paper also provides a medical adaptation to the automotive ID-Link process workflow into a feasible seven-step model from patient consent, doctor interaction, ID check, commercial use, payment, and profit share. The ID-Link is built in four architectural layers business, functional, data, and technological layers.

Guo et al. (42) criticize the processes that digital health innovators follow to draw results for their solutions and implementations. The authors also emphasize a lack of implementation when it comes to digital health solutions, and therefore it is not easy to draw any evidence-based results. The study analyzed some of the major digital health solutions implementations against selecting nonexclusive relevant regulatory standards and the methodologies that innovators adopted in evaluating their solutions. Nevertheless, the authors acknowledge that the innovators do not create barriers and that innovators are stuck in the “no evidence, no implementation—no implementation, no evidence” paradox in digital health. The authors suggest that approaches, such as simulation-based research, can generate higher-quality, lower-cost, and more timely evidence.

Affinito et al.’s survey (43), in contrast, is to understand the digital means that physicians are using to engage with their patients and the effect physicians perceive on clinical health outcomes. The survey results suggested that the main success factors in achieving patient empowerment with digital tools and improving health outcomes are clinical evidence and actual users’ (patients and caregivers) involvement in designing the digital solutions. The study concludes that the use of digital tools would do improve patient empowerment. Nevertheless, to date, there is no evidence of an improvement in health outcomes.

Having established that there is no evidence to support that patient outcomes improve with the adherence to using digital tools, Angeline and Sharon (44) conducted a study to investigate whether the level of digital literacy among healthcare staff is to be blamed. This study demonstrated that the majority of staff showed confidence in using ICT. However, it is understood that the location of the study (Australia) might have affected the results of the study and that we should anticipate other results in other territories.

Electronic Health Records for Clinical Research EHR4CR is a European project that aimed to enhance the patient-centric trials by developing a platform that allows access to existing patients’ EHR (45) making the project a lot similar to the DHDM research project. Except that it does not handle the patient compensation for the usage of his or her EHR data. Dupont et al. (46) is a study that assesses the financial results of the project. The study compared EHR4CR to existing practices and concluded that EHR4CR solutions seem to be cost saving for primary sponsors of clinical trials. The study results suggest that the potential for savings would increase with the broader adoption of EHR4CR solutions in Europe and beyond. The results, in turn, suggest that a medical data marketplace where patients can sell access to their EHR records for their own benefit would in the long run save cost in industrial and clinical trials.

In a paper, Timo and Harri (47) aimed to develop an Ecosystem Evaluation Framework (EEF) for understanding the chances of survival of a digital business platform. The authors described the EEF model in six parameters (the platform, the problem that the platform is trying to reduce, the purpose of the platform, the ecosystem, the transactions enabled by the platform, and the revenue model of the platform). The authors highlighted the importance of considering the compensation model, which perfectly matches the goals of our study. They relate missing the incentive component to be the main reason for the failure of the regional health information system RHIS to reach critical masses in the Pirkanmaa region in Finland, where they applied their model.

Alina and Jose Luis (48) discussed what the authors called a FAIR marketplace. They identified the attributes for the data to be Findable, Accessible, Interoperable, and Reusable, and this is where FAIR came from. The authors presented an architecture that accommodates layers to gather information from patients, care providers, and other platforms such as EHR4CR.

Credit score has always been the biggest constraint to the DHDM research. In almost every survey, poll, or even friendly chatting, this issue has been raised. People wonder if the project will make them exposed as the credit score does with their finances. People are always worried about being denied or paying more for services they are now getting without significant exposure to health history, such as renewing motor insurance. They have concerns about higher premiums once the insurance knows more details about their health or, worse, being denied services if they do not allow access to their records as it happens with the credit score.

However, credit scoring architecture is a perfect example of data aggregation and permissioned sharing from a techno-commercial perspective. Dumitru and Gatti (49) discussed the constraints and the opportunities related to sharing health data and the usage of the data for credit scoring purposes. The authors have proposed an architecture for a trusted data marketplace that can be very useful to act as the weighing system in the DHDM project. The weighing system is what calculates the contribution of each EHR into an entire data set. It shall be used to equitably distribute the payments from medical researchers between the data owners in a way that incentivizes the EHR based on their commitment to wellbeing and their commitment to keeping the EHR up to date.

A study conducted by Roman and Stefano (50) is a practical example of capitalizing on the successful credit scoring model in calculating the weight/value of every EHR entry. The weighing component has massive value in a fair distribution of wealth between EHR owners based on the contribution of each entry and each EHR in the research to which the wealth has been paid.

Ryuji’s study (41) is another practical example that could benefit the DHDM research project. It provides a workable model of commercial exchange of funds against data that capitalize on already implemented techniques in the automotive industry.

## CONCLUSIONS

Although billions of dollars are spent on making the current health data management systems more efficient, data sharing remains an elusive goal in a health sector. As GDPR has introduced a paradigm shift on data ownership and control along with blockchain-like technologies, providing the technological capability of decentralized data management; it is the right time to change the underpinning incentivization model through a DHDM-like open market model.

Based on this review, it is evident that blockchain-based solutions like MedRec (23) can be implemented as a separate layer and integrated with native databases through application programming interfaces (APIs) without perturbing native data management systems and culture, which will definitely benefit the technology adaptation process. Moreover, being open-source, MedRec-like solutions will play a significant role in secure data collection from existing data management systems and combining an aggregated EHR under the patient’s control. Smart contract and IPFS/cloud storage systems will provide patients the control to securely grant access over different types and duration of de-identified data. The review demonstrated different proposals for data access and secure sharing between data producers and consumers. However, further studies need to be performed on digital data reproduction and how to secure the producer rights if the consumer reproduces the data beyond their consent. Further studies are also needed to identify how to adapt the data sharing process with varied regulations across different geographical jurisdictions and time.

The shared economy-based incentivization model that is deemed most appropriate for the DHDM context also needs to be evaluated extensively. Although a company like Airbnb has demonstrable economic benefits for both provider and consumer, sharing personal health data may have a different social and emotional context than personal accommodation. Despite these concerns, it is almost certain that an open marketplace will introduce competition to produce and impetus to share high-quality data according to consumer demand. This in turn will facilitate researchers and medical practitioners to readily access data according to their requirements.
